# Practical design of variable fractional-order capacitors with a single tuning feature using field effect transistors and variable capacitance diodes

**DOI:** 10.1038/s41598-025-07319-5

**Published:** 2025-07-01

**Authors:** Roman Sotner, Chloe Black, Jan Jerabek, Todd Freeborn, Simon Colburn, Marek Svoboda

**Affiliations:** 1 Faculty of Electrical Engineering and Communication, Brno University of Technology, Technicka 3082/12, Brno, 616 00 Czech Republic; 2 Department of Electrical Engineering, Faculty of Military Technology, University of Defence, Sumavska 4, Brno, 602 00 Czech Republic; 3 Department of Electrical and Computer Engineering, The University of Alabama, Box 870286, Tuscaloosa, 35487 USA

**Keywords:** Adjustability, Constant phase element, Fractional-order, MOSFET, Pseudocapacitance, Tunability, Varicap, Electrical and electronic engineering, Electronics, photonics and device physics, Techniques and instrumentation

## Abstract

This paper presents two discrete circuit solutions for realizing passive, electronically adjustable constant-phase elements, specifically half-order capacitors with a –45° phase shift. Fractional-order capacitors with electronically adjustable pseudocapacitance are especially useful for designing tunable filters and oscillators. The ability to adjust pseudocapacitance electronically and continuously is a major improvement over traditional passive solutions. Their pseudocapacitance can be controlled by a DC voltage, allowing key parameters like the cut-off or oscillation frequency to be tuned. Two presented design approaches differ in accuracy, tuning range, and signal-handling capability. Both solutions maintain a constant phase over one frequency decade, with a phase ripple within ± 2°. The tuning range spans from hundreds of Hz to several MHz. Presented solutions allow pseudocapacitance tuning in range of hundreds of nano F/sec^0.5^ (with varicaps) and tens of micro F/sec^0.5^ (with MOSFETs). The MOS-based circuit offers a tuning ratio of 7 but shows a 19% deviation between simulation and measurement. It also suffers from notable nonlinearity, with undistorted operation limited to signal levels up to 20 mV peak-to-peak. The varicap-based solution achieves a tuning ratio of 5, with high accuracy (up to 6% error), and handles input signals in the hundreds of mV with acceptable distortion. PSpice simulations and laboratory measurements confirm the performance of both designs.

## Introduction

Fractional-order passive elements have increasingly attracted the attention of researchers and designers in recent decades^1^. This class of elements has electrical impedance characteristics between traditional passive elements (e.g. resistors, capacitors, and inductors). For this reason, these elements are often referred to as fractional-order capacitors or fractional-order inductors^1,2^ when the device impedance is between a resistor and capacitor or resistor and inductor, respectively. Focusing on a fractional-order capacitor, which is also widely referred to as a constant phase element (CPE), the impedance of this element is given by: *Z*_CPE_ (s) = 1/(s^α^*C*_α_), where α is the fractional-order (0 < α < 1) and *C*_α_ is the so-called pseudocapacitance (physical unit F/sec^1−α^). The theoretical phase of this element is within the range −90° < *ϕ*_α_ < 0° and is related to the order such that:* ϕ*_α_ = −α⋅90°. Notice that it is the fractional-order which sets the impedance phase and that this is independent of frequency. The special case when α = 0.5 (*ϕ*_α_= −45°) is referred to as a half-capacitor in this work. It is important to note that the language fractional-order is in reference to the electrical impedance relationship of these elements being defined by fractional-order differential equations^3^, the branch of mathematics concerning differentiation and integration to non-integer orders.

The use of fractional-order circuit models to represent the measured experimental impedance of materials and devices, such as solid-state devices and liquid substances^2^, is an expanding field of research. Many of these research efforts are focused on characterization of these systems and identification of circuit models (and circuit model parameters^4,5^) to accurately represent the system or device. The underlying motivation is linked to using changes in circuit model parameters (associated with underlying changes in electrical impedance) to understand the device structure or device changes over time or conditions. This motivates the exploration of fractional-order models for food quality assessments (fruit^6^, drinks/liquids^7–9^, , storage and degradation^10–12^), characterizing healthy and pathology tissues in biology and medicine^13–16^, and supporting agriculture and plant cultivation^17,18^.

The use of fractional-order circuit elements to support modeling and design applications is usually referred to as the field of fractional-order circuits and systems^19^. Within this field, when focused on using these elements in the design and realization of electrical circuits for signal conditioning, these circuits are referred to as fractional-order filters^20–24^. The unique features of a fractional-order filter (in comparison to its integer order counterpart) are the independent control of attenuation and phase slope, which also has implications for filter delay characteristics. Similarly, these fractional-order components enable continuous and arbitrary phase shift between generated sinusoidal waveforms for multiphase oscillators^25–30^. These solutions highlight the important theoretical contribution of a fractional-order mindset in circuits and system design^28–30^. For example, recent work by A.S. Mohapatra et al.^30^ combines both fractional-order impedance modeling and fractional-order circuit design to advance knowledge and applications of fractional-order impedance measurements using oscillator circuits.

While it is possible to design fractional-order circuits with theoretical fractional-order elements, their realization requires using physical elements. However, fractional-order elements are not yet commercially available and are limited to research groups with the expertise and facilities to fabricate them^31–34^. Until these devices become widely available fractional-order circuit designs require an approximant to be realized. Approximants can be broadly classified as active or passive based on if they use components that require external power for their operation. A common passive approximant uses resistor-capacitor (RC) ladder structures^22–27,35,36^ to realize a device that emulates the fractional-order impedance of the designed element^37,38^. With this approach, the resistor and capacitor components are used to realize poles and zeros (roots) of the target impedance function. Active designs use a similar approach but utilize components such as operational amplifiers, operational transconductance amplifiers, and current conveyors^19–21^. This approach has higher flexibility based on features that can be adjusted using a tuning voltage or current. However, many tuning features have to be modified simultaneously and the circuitry to achieve this is extensive^20^, ^21^. There are also active design approaches that utilize so-called bilinear sections (two-port networks or impedances of independently adjustable zero and pole locations) to realize a target function by cascade of these sections^39–41^. But this approach also brings complexity to the resulting circuitry.

**Table 1:** Comparison of basic features of recent and typical passive fractional-order two-terminal devices (including liquids, solid-state and RC, RL approximants).

References	Solution	Total number of elements	Experimentally tested frequency band (number of decades of valid operation)	Available C_a_	Targeted phase (α)	Electronic tunability of C_a_	Insignificant influence of time stability of parameters	Design and fabrication costs^+^
^31^	Liquid	-	20 kHz→200 kHz (1)	N/A	−10 → −12° (0.11 → 0.14)	No	No	High
^32^	Liquid	-	100 Hz→1 MHz* (1)	N/A	various**	No	No	High
^33^	Solid-state	-	20 Hz→2 MHz* (5)	N/A	−30° (0.33)	No	No	High
^34^	Solid-state	-	200 Hz→5 kHz (1)	N/A	−37° (-0.41)	No	Yes	High
^35^	RC, RL	16	10 Hz →1 MHz*** (5)	Any***	Any***	No	Yes	Low
^36^	RC	12	10 mHz→1 kHz (4)	20 mF/sec^0.5^	−45° (0.5)	No	Yes	Low
Proposed								
Figure 6	MOS(*R*_DS_) + C	8	500 Hz→100 kHz (1)*	15→109 µF/sec^0.5^	−45° (0.5)	Yes	Yes	Low
Figure 12	R + varicap	13	8 kHz→5 MHz (1)*	138→654 nF/sec^0.5^	−45° (0.5)	Yes	Yes	Low

C_α_ – pseudocapacitance; α – order; *one decade of operability at frequencies in indicated range; **several different CPE samples of various C_α_ and α tested; ***frequency range fulfilled with each CPE, various C_α_ and α possible for set of novel RC values; ^**+**^the fabrication costs tend to be high (several hundreds of Euros or more) when specialized chemicals, materials, or CMOS processes (chip design) are involved. Conversely, costs remain low when readily available, off-the-shelf components are used – typically limited to just a few Euros.

For interested readers a recent survey of methods to approximate fractional-order elements is available^42^. For a brief comparison, Table 1 presents typical features and physically allowed features of recent approximates. Emerging themes from this comparison are: (a) liquid and solid-state solutions^31,32,34^ may have a very large frequency band, but the operational frequency range of constant phase (defined by allowed phase error/ripple Δ*ϕ*_α_) is limited to approximately one decade, (b) many solutions^31–34^ do not prioritize pseudocapacitance values, (c) electronic adjustment of pseudocapacitance is not possible and studied in passive solutions, (d) CPEs can be influenced by long-term stability of material features^31–33^, (e) the fabrication costs may be high for complex technological process^31–34^ in comparison with passive elements-based CPE approximants^35,36^.

## Motivation and goals

From the review of recent works and available approaches to realize fractional-order elements, it is clear that passive designs with features to easily adjust the pseudocapacitance are not available. This limitation is expected to decrease wider interest in using fractional-order elements because their theoretical improvements cannot be easily realized or tuned during implementation of a design. This motivates this work, which has the goal to design and validate a practical implementation of a variable fractional-order capacitor (α = 0.5) with a single tuning feature to adjust the pseudocapacitance. The target goals and driving expectations for this practical implementation include: (a) propose and verify features of simple RC-based CPE allowing single voltage electronic adjustment of pseudocapacitance, (b) operational bandwidth (validity of defined phase ripple) reaching at least one frequency decade, (c) frequency range at least up to 100 kHz, (d) using grounded topology of CPE because of its simple implementation having low influence of stray capacitances (and other parasitics), (e) linearly operating element creating insignificant distortion and waveform degradation to applied signals, (f) possibility to use low-cost solution and components available on the market for designers in the field.

The following sections outline the measurement setup utilized to collect experimental data throughout this work (Sect. 3), the design approaches for scaling and parameter adjustment (Sect. 4), proposed solutions using MOSFETS (Sect. 5) and varicaps (Sect. 6) as adjustable elements with their experimental validation, and the wider discussion of this work in the context of this field (Sect. 7), and overall conclusion (Sect. 8).

## Measurement setup

**Fig. 1:**
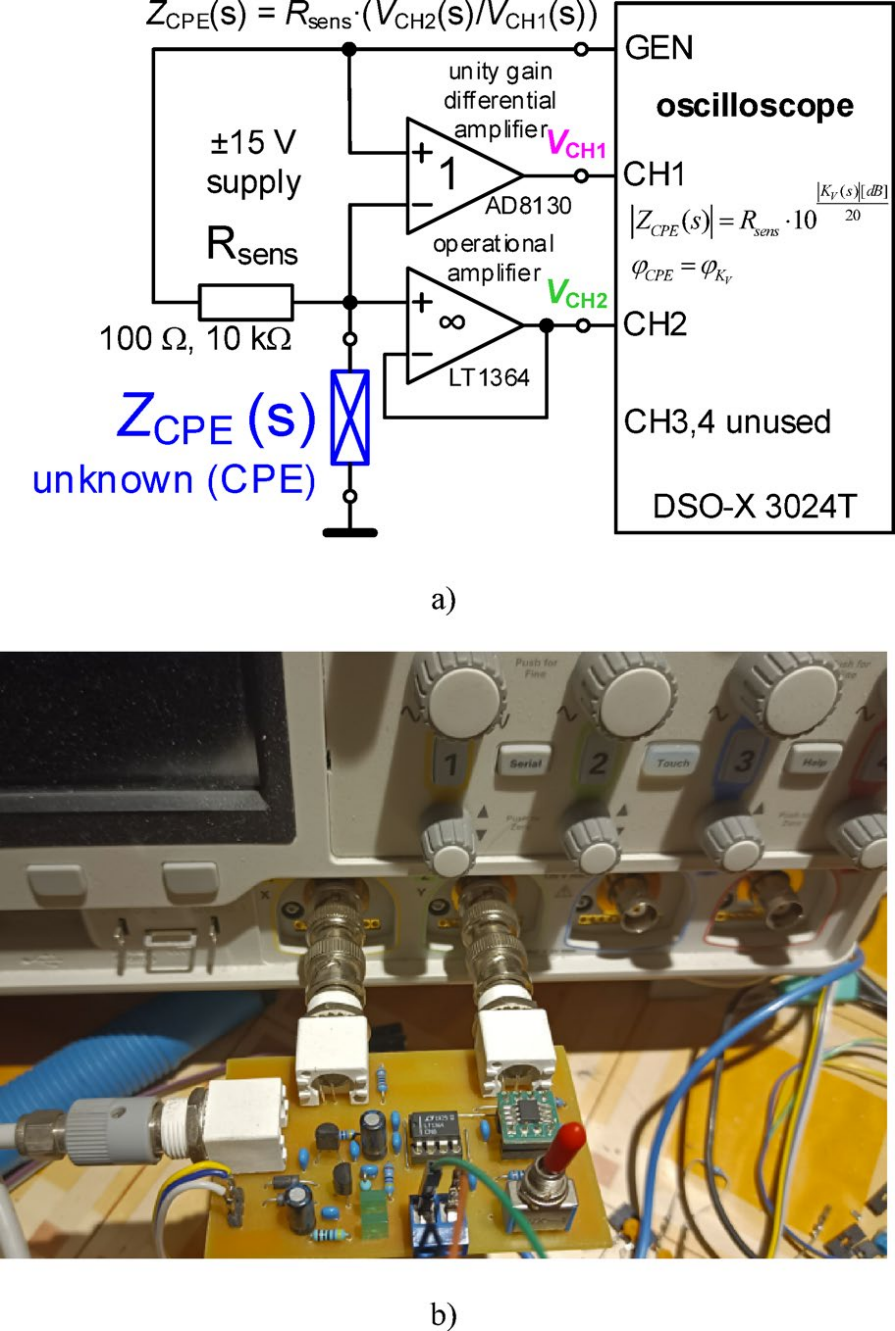
The (a) high-level read-out interface design and (b) physical implementation of the interface connected to a Keysight DSO-X 3024T oscilloscope.

To measure the electrical impedance of the proposed fractional-order designs in this work a custom read-out design interfaced to an oscilloscope was utilized. The high-level design and physical implementation of this read-out interface is given in Fig. 1(a) and (b), respectively. This interface is designed to measure impedances in the frequency range of 10 Hz to 10 MHz and is compatible with a Keysight DSO-X 3024T oscilloscope. In this setup the unknown impedance (*Z*_CPE_(s) in this case) is a component in a resistive divider. The voltage across the sensing resistor (*R*_sens_) and unknown impedance are both captured by the oscilloscope as shown in Fig. 1(a). The resulting impedance is expressed as: *Z*_CPE_(s) = *R*_sens_⋅*K*_V_(s), where *K*_V_(s) = *V*_CH2_(s)/*V*_CH1_(s) is the voltage transfer between channels. For these measurements, the oscilloscope is configured to generate a sinusoidal voltage at the GEN output and the frequency is swept from a start to end frequency. The collected measurements of this transfer voltage are reported in dB and require conversion to impedance amplitude using: |*Z*_CPE_(s)| = *R*_sens_⋅10^(|KV(s)| [dB]/20)^. The phase (argument) characteristic φ_CPE_ (φ_α_) of the impedance directly corresponds with the phase characteristic of *K*_V_(s), i.e. φ_KV_. The choice of *R*_sens_ does influence the voltage magnitude and values of 100 Ω (suitable for small values of impedances) and 10 kΩ (for large scale of measured impedance) were used in this work based on the expected magnitude of *Z*_CPE_(s) measurements.

## CPE design approach

To realize an approximant with tunable pseudocapacitance we propose the substitution of either MOSFETs or varicaps for elements within a traditional RC ladder structure. These elements have voltage-dependent resistance (MOSFETs) or capacitance (varicaps) which can be used to adjust the overall impedance of the ladder (and representative pseudocapacitance). Further, these components are both widely available and affordable making them attractive options for a practical, easily realizable design. However, prior to this substitution the following section outlines the design of the RC ladder structure.

### Theoretical explanation of initial CPE features and scaling

An RC approximant of a CPE can use several parallel or serial sections of RC elements^37,38,42^. Generally, the greater the number of sections, the greater the approximated impedance accuracy. The design of the approximant requires that its zeros and poles are placed at frequencies appropriate to shape the impedance characteristics to approximate the impedance of the CPE, *Z*_CPE_(s)^35,37–41^. However, these pole/zero locations are mutually dependent on all circuit elements in the structure. This requires pairs of values to be recursively calculated from previous sets until the design requirements are achieved (which increases in complexity with increasing numbers of segments). As a result of this iterative approach, it is possible for various values of RC elements to form the complete impedance function. This mutual dependence complicates (or can even prevent) tunability of a CPE because each single value variation has global impact on all poles and zeros.

**Fig. 2:**
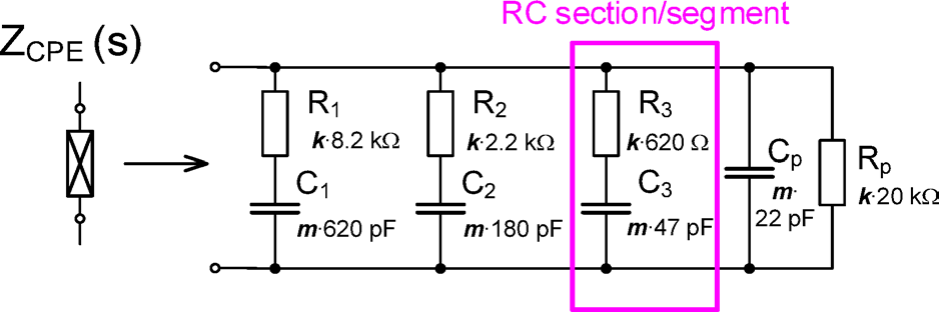
A sample RC ladder structure to realize a CPE approximant with scaling factors (*k*,* m)* used for tuning C_α_.

**Fig. 3:**
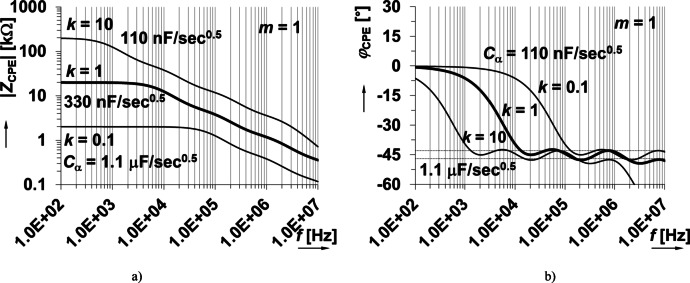
Ideal impedance (a) magnitude and (b) phase responses of approximated CPE using RC ladder structure with *m* = 1 and *k* = 0.1, 1, 10.

As an example, the RC ladder structure in Fig. 2 represents an approximant CPE formed by the parallel combination of serial RC segments^37,38^ with the following features: α = 0.5 (φ_a_ = φ_CPE_ = −45°), *C*_α_ ≅ 350 nF/sec^0.5^ (actually 330 nF/sec^0.5^, after rounding to E12 series), frequency range from 30 kHz up to 5 MHz (more than two decades), and phase ripple Δφ_α_ = Δφ_CPE_ = ± 2°. Note that the design and element values in Fig. 2 were selected intentionally to be close to the feasible ranges of the electronically adjustable substitutes (MOSFETs and varicaps) that will be used for later *C*_α_ tunability in this work. The magnitude and phase of this RC ladder structure is given in Fig. 3(a) and (b), respectively, as the thickest solid line.

**Fig. 4:**
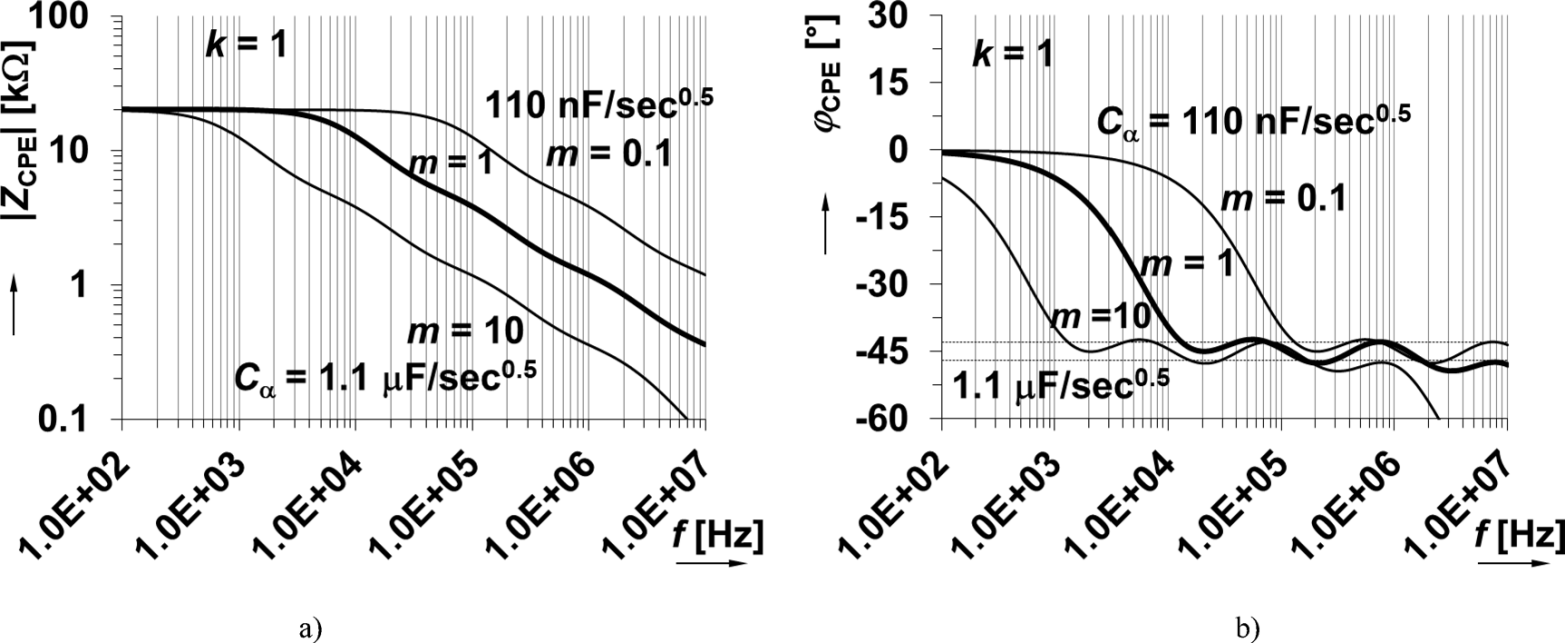
Ideal impedance (a) magnitude and (b) phase responses of approximated CPE using RC ladder structure with *k* = 1 and *m =* 0.1, 1, 10.

Note that in Fig. 2 the resistor and capacitor values have scaling factors *k* and *m*, respectively, which can be used to change the *C*_α_ value (where *k* = *m* = 1 yields the unscaled case) of the approximant. Both factors have intentional impact on *C*_α_ with *k* impacting the impedance magnitude (*R*_CPE_ value when frequency approaches 0 Hz) and operational frequency range. The factor *m* impacts the operational frequency range, but *R*_CPE_ remains unchanged for all values of *m*. To demonstrate how *Z*_CPE_(s) is scaled, the impedance was calculated when *k* and *m* were varied from 0.1 up to 10 (*k*_max_ = *R*_maxi_/*R*_mini_, *m*_max_ = *C*_maxi_/*C*_mini_, where i = 1, 2, 3, … is number of specific RC segments). From Fig. 3(a), notice that increasing *k* (when *m* = 1) increases the magnitude across all shown frequencies and increases operational frequency range. Similarly, increasing *k* (when *m* = 1) shifts the approximated phase frequency band to lower frequencies in Fig. 3(b). Similar shifts are observed in Fig. 4 for increasing *m* (when *k* = 1). This demonstrates that by scaling the RC ladder values the impedance magnitude and phase can be transformed. From review of the impedance magnitude in Figs. 3 and 4, the theoretical value of *C*_α_ ranged from 110 nF/sec^0.5^ to 1.1 µF/sec^0.5^ for this set of *k* and *m* variations. This suggests that 2 decades of *k* and *m* change are required for one decade of *C*_α_variation (for this specific ladder and component values). As a summary, both parameters influence the pseudocapacitance value. Parameter *k* corresponds to variation of resistor values and *m* to variation/scaling of capacitor values. We can observe that *k* has influence on the impedance for frequencies close to 0 Hz. On the other hand, the parameter/scale *m* does not have effects on the DC resistance of CPE. We can see that both parameters are shifting the operational bandwidth in the horizontal (frequency) direction. From the analysis shown in Figs. 3 and 4 we can see that change of the pseudocapacitance by one decade requires change of the *k* and *m* by two decades (therefore *k* and *m* between 0.1 and 10 were used in demonstrations, to be able to present one decade shift in case of pseudocapacitance. The initial design values of Valsa’s prototype in Fig. 2 without scaling are obtained for *k* = *m* = 1.

Component scaling is an effective approach for *C*_α_ adjustment in this topology but does have limitations. Specifically, the element values of the RC segments are distributed across several decades. Therefore, an adjustable device to use as a substitute for either R or C in the ladder will require a wide range of operating values and needs identical scaling changes for identical driving voltages. In the context of the sample shown, a replacement device should be available with values across several decades *R*_mini_→*R*_maxi_ (*C*_mini_→*C*_maxi_) and adjusted by the same driving voltage *V*_set_Cαmin_→*V*_set_Cαmax_ in each segment, e.g. 1 kΩ→10 kΩ, 10 kΩ→100 kΩ, etc. all by the same voltage (e.g. 1 V → 10 V).

### Single-parameter control of pseudocapacitance

The adjustment of resistor and capacitor values in each section of the topology in Fig. 2 for the demonstrated *k* and *m* is not practical by standard electronically adjustable passive elements because of the wide range of values (e.g. 0.82 kΩ → 82 kΩ for *R*_1_). There are no electronically adjustable equivalents of linearly operating resistors with an operating range over 2 decades for this particular frequency bandwidth^43^. Another challenge that needs to be addressed is how to use only a single tuning parameter (e.g. one tuning voltage) to adjust the pseudocapacitance. Therefore, a design approach to select appropriate component values to meet the design requirements and that are within the tuning range of the variable components is needed.

To address this challenge, we propose a design approach that unifies the value of resistors and capacitors across segments (with the unified value used for single-parameter tunability) and scales the non-unified component values as required to meet the impedance requirements. This approach limits the usable operational range of the approximated CPE to a single frequency decade only (demonstrating the trade-off of increasing tunability at the cost of operational range).

**Fig. 5:**
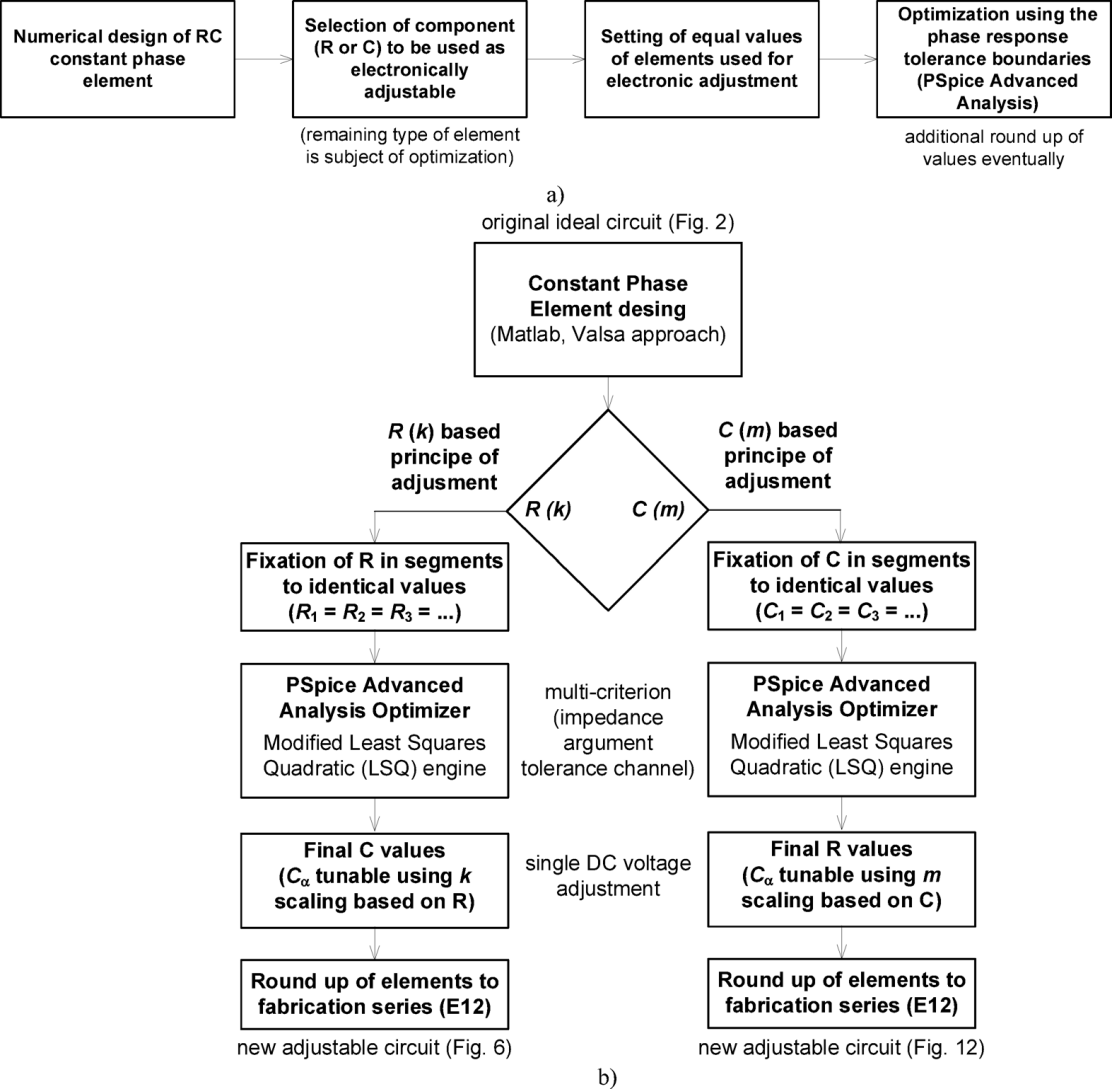
Design methodology of electronically adjustable CPE: (a) general methodology and the design flow, (b) high-level design procedure to revise an RC ladder structure to realize a single-feature tuning topology.

The high-level design process is described in Fig. 5. Starting with an RC ladder circuit, with topology and parameter values selected using established methods (such as that proposed by Valsa^38^), the values of resistors or capacitors are then fixed to a user-selected value in each segment. Next, a numerical optimization procedure is implemented to solve for values of the non-unified components (e.g. capacitors when resistors are fixed or vice versa). In this work, the optimizer tool of the PSpice Advanced Analysis module was utilized and configured to solve for component parameter values that would yield impedance phase values at a set of discrete frequencies within the target ripple (e.g. −45**°** with +/− 2**°** ripple). Finally, after identifying all values they are rounded to the nearest E12 value to support the rapid realization of the design. The use of this approach and the resulting values that it yields are demonstrated for both approaches (e.g. unifying resistors and unifying capacitors) in the following sections.

## MOSFET-based controllable half-capacitor

**Fig. 6:**
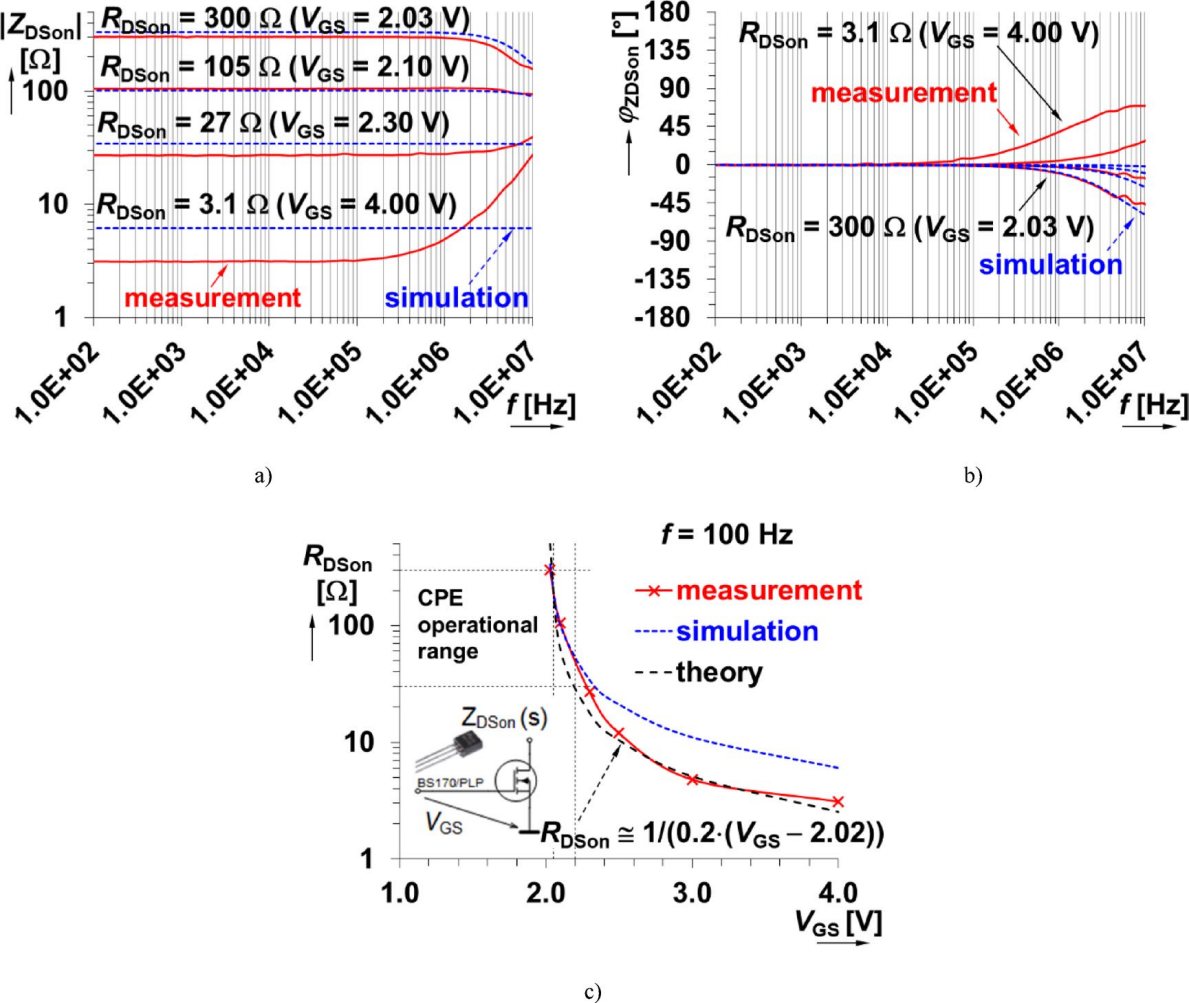
Experimental characteristics of MOS-based impedance (BS170) in triode regime: (a) impedance magnitude plots, (b) impedance phase plots, (c) dependence of low-frequency drain-source resistance on gate-source voltage.

To demonstrate the design approach to realize a tunable CPE approximant using *k* scaling, components with an adjustable resistance are required. One type of component with this behavior is a MOSFET operating in the triode regime. To demonstrate the variable impedance of a BS170^44^ device, experimental measurements of the drain-source impedance (*Z*_DSon_(s)) for gate voltages from 2 V to 4 V were collected. These magnitude and phase measurements of BS170 are shown in Fig. 6, demonstrating an on-resistance from approximately 3 Ω to 300 Ω for this range. While the measured transconductance, *g*_fs_ = 200 mA/V^2^, was lower than the datasheet this is not unexpected based on the large parameter tolerance, complex nonlinearity for *R*_DSon_ ≅ 1/(*g*_fs_⋅(*V*_GS_ − *V*_th_))), and particular threshold voltage *V*_th_ = 2.02 V (from experimental evaluation). For reference, the nominal (typical) parameters of the BS170 device relevant to our purposes, according to the datasheet^44^, are: *g*_fs_ = 320 mS and *V*_th_ = 2.1 V. However, the variations in *V*_th_ due to fabrication process and temperature effects are expected to fall within the range of 0.8 V and 3 V – more than a 50% deviation.

The frequency dependent impedance of this device is clear from the measurements in Fig. 6 with changes in both magnitude and phase as frequencies approach 10 MHz. From the datasheet, the inter-terminal capacitances *C*_GS_ and *C*_DS_ are quite high (tens of pF), which is the expected cause of the decreasing magnitude at frequencies above 1 MHz (when *R*_DSon_ > 100 Ω). The device inductance is expected to be the source of the increasing impedance magnitude in Fig. 6(a) at frequencies above 1 MHz (when *R*_DSon_ < 100 Ω). The inductive and capacitive high frequency behaviors are also observed in the phase of Fig. 6(b). This highlights that this device does not behave as an ideal resistor, which will have impacts on the frequency bands over which it can be used for a CPE approximation. Note that all parameters indicated in the figures are the measured results unless otherwise stated. The simulated low frequency (100 Hz) drain-source resistance for varying gate-source voltage is shown in Fig. 6(c). Note that the transistor has a very large difference between simulation and experiment/theory for *V*_GS_ > 2.2 V, but clearly demonstrates a voltage-dependent resistance that can be used for our design.

**Fig. 7:**
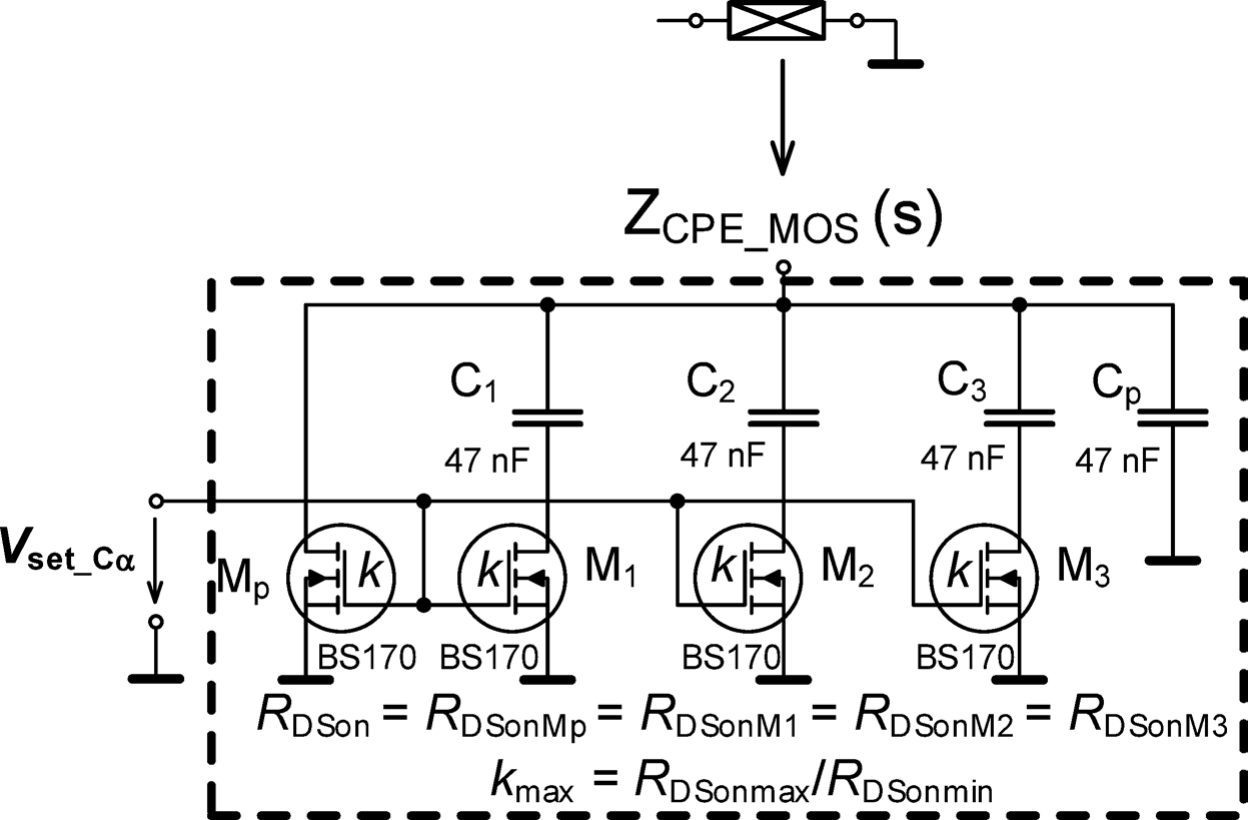
The MOSFET-based adjustable CPE to realize a half-capacitor with *C*_α_ = 330 nF/sec^0.5^ tunable using a single voltage.

Our design of a tunable MOS-based CPE (Fig. 7) starts with the initial circuit shown in Fig. 2 with nominal value *C*_α_ = 330 nF/sec^0.5^ and *k* = *m* = 1. Using the approach outlined in Fig. 5, all resistors were fixed to 100 Ω (because *R*_DSon_ = 100 Ω is a feasible value with the MOSFETs). Next, the criterion frequencies from 10 kHz to 100 kHz (10 kHz, 30 kHz, 60 kHz, 100 kHz) were selected for the optimizer with phase band from −47° and −43° (e.g. −45° ± 2°). The specific frequency band was selected because the approximation in this frequency band (shown in Figs. 3 and 4) well represented the target impedance. Next, using the optimization tool of PSpice Advanced Analysis module yielded the capacitor values of 51 nF, which were reduced to 47 nF (to compensate for the MOSFET *C*_DS_ contribution and select the closest E12 series value). While the capacitors have the same value, this is unique to this particular case and not a general rule.

**Fig. 8:**
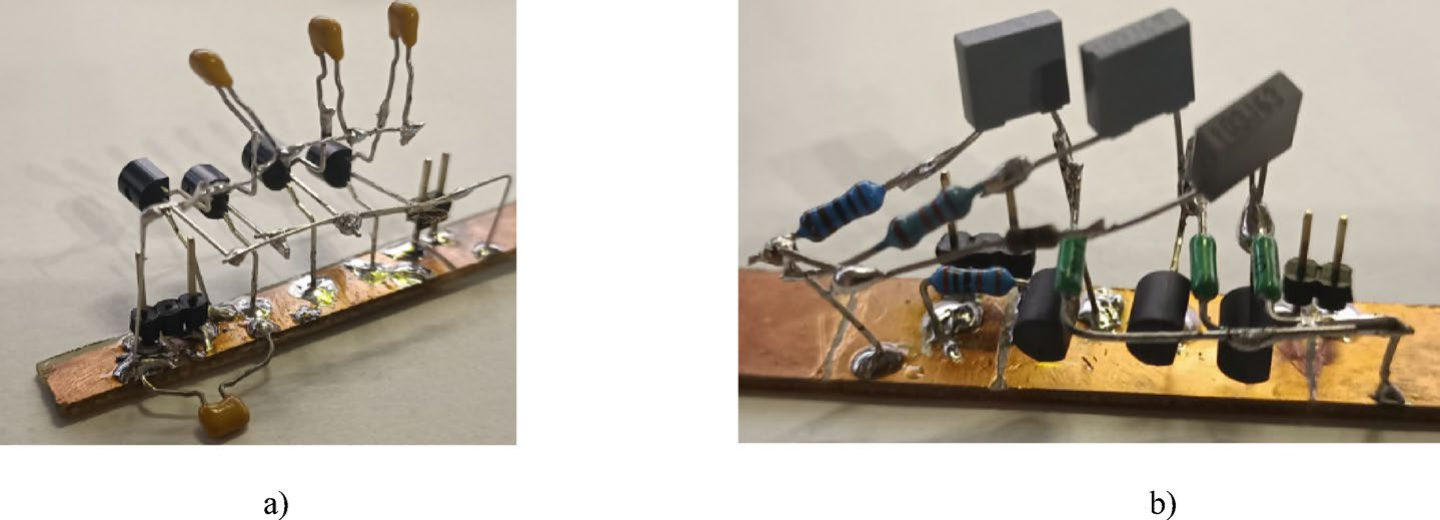
Physical realization of the adjustable CPE designs using the (a) MOS-based, (b) varicap-based methods.

**Fig. 9:**
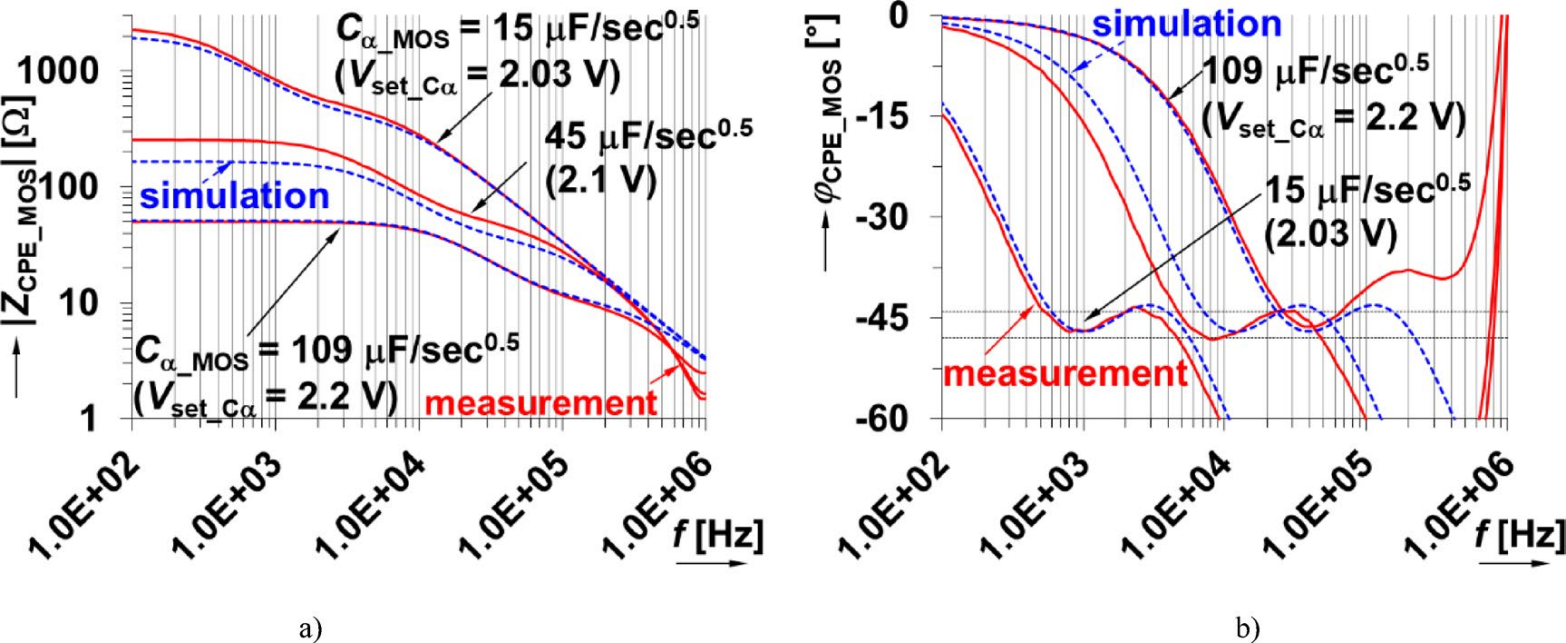
Measured and simulated impedance (a) magnitude and (b) phase of proposed MOSFET adjustable CPE.

**Fig. 10:**
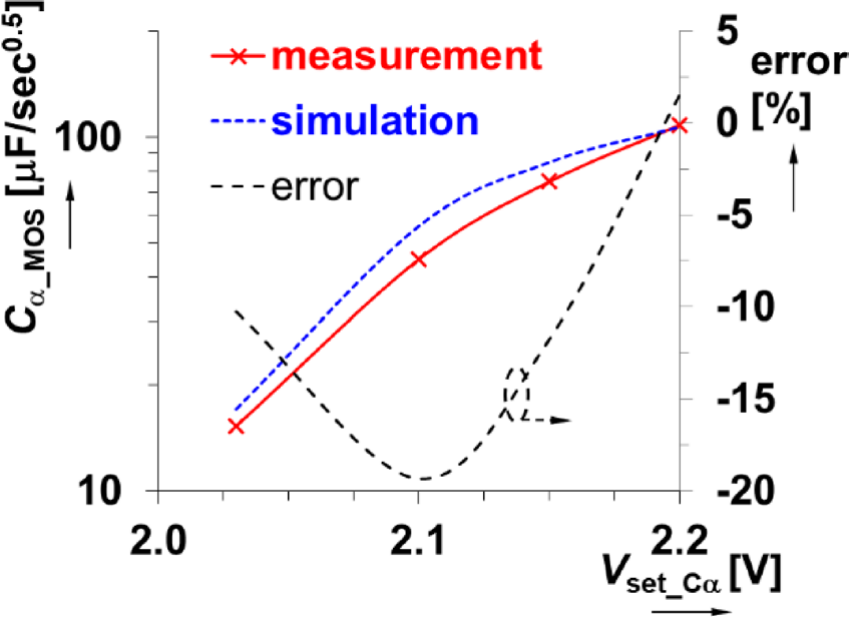
Dependence of pseudocapacitance of MOSFET_based CPE design on the driving voltage.

The physical realization of this design is given in Fig. 8 using discrete components assembled on a copper plate to minimize parasitic capacitances (compared to a standard printed circuit board or breadboard realization). The experimental impedance was measured (using the sensing readout and experimental interface described in sec3) for applied gate source voltages of 2.03 V, 2.1 V, and 2.2 V. The experimental results compared with PSpice simulations of the design are shown in Fig. 9 for both magnitude and phase. The experimental maximum value of *C*_α_MOS_ is larger than the nominal (*C*_α_ = 330 nF/sec^0.5^), which is attributed to the significantly lower resistor values (when compared to the ideal nominal case). The *C*_α_MOS_ value is strongly associated with the fixed parameter values of the CPE (*R*_DSon_ range in this case). The experimentally obtained *C*_α_MOS_ range (Fig. 10) spans from 15 µF/sec^0.5^ to 109 µF/sec^0.5^ when *V*_set_Cα_ ranges from 2.03 V to 2.2 V. This is very close to the simulation range of 17 µF/sec^0.5^ to 107 µF/sec^0.5^. These simulation and experimental results confirm the tunability of the *C*_α_ value by adjusting only one single value of this design. Overall, this design has a center value 45 µF/sec^0.5^ and valid bandwidth between 4.6 kHz and 49 kHz (meeting the 1-decade operational frequency goal of this work). The larger differences between simulation and experimental results (greater than 10%) shown in Fig. 10 are primarily due to significant variations in the parameters of real MOS transistor (BS170), particularly the threshold voltage, when compared to the PSpice model. Additionally, there is a notable difference between the behavior of the nominal simulation model and the experimentally tested sample illustrated in Fig. 6. These variations in static parameters depend on fabrication tolerances and temperature effects, as provided in datasheet^44^. However, the inaccuracies between measurement and simulation are less significant for practical applicability. Based on Fig. 6 (behavior of simulated, measured and theoretically described MOS element), the deviation from theory will be significantly lower (units of %) than in comparison to simulation. The suitability of the device BS170 for high-frequency design is very limited by the gate-source, gate-drain and drain-source parasitic capacitance (up to 40 pF^44^) creating in the CPE segments an additional undesired zero-pole frequencies disturbing overall phase response of the CPE (above 100 kHz).

While the physical realization aimed to reduce parasitics associated with the overall assembly, there are still parasitic influences of the BS170 above 100 kHz. These influences are observed in both magnitude and phase deviations of the experimental values from the simulations above 100 kHz. This is especially clear in the phase data of Fig. 9(b) for *V*_set_Cα_= 2.2 V where the phase ripple is not fulfilled in the operational band and deviating significantly from the simulated expectations.

**Fig. 11:**
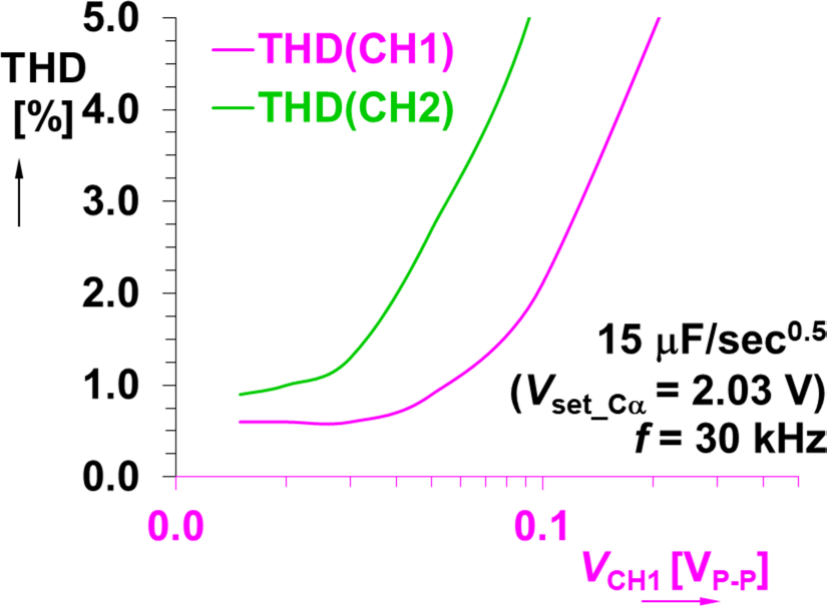
The dependence of THD on voltage levels measured in experimental setup (Fig. 1) for MOS-based CPE.

Another important consideration when using the MOS device is the high nonlinearity and changes of regime of operation when *V*_DS_ > > 0 V. As a result of this nonlinearity, the total harmonic distortion (THD) can rapidly increase over different operational ranges. To evaluate the THD of the adjustable CPE, experimental values were collected using the sensing readout (shown in Fig. 1). For this analysis, a testing frequency 30 kHz was selected because it is in the middle of the operational bandwidth of the CPE (configured as *C*_α_MOS_ = 15 µF/sec^0.5^). The THD of the voltages of channel 1 (voltage across the sensing resistor) and channel 2 (voltage across the CPE) are given in Fig. 11. The THD value starts to increase rapidly over 1% when the applied voltage level is larger than 100 mV (green axis and green trace). This suggests that this MOS adjustable CPE with controllable value of *C*_α_will have the best performance for amplitude levels up to several tens of mV.

## Varicap-based half-capacitor

To demonstrate the design approach to realize a tunable CPE approximant using *m* scaling, components with a adjustable capacitance are required. One type of component with this feature are voltage adjustable capacitors (or varicaps). As an example of this feature, the varicap BB112^45^ has a capacitance, *C*_var_, ranging from approximately 800 pF to 30 pF for DC driving voltages (reverse bias voltage) *V*_var_ from 0 V to 8 V. This driving voltage range is very wide in comparison with the adjusted capacitance range, but it makes the design more stable and accurate than the previous MOS-based CPE.

**Fig. 12:**
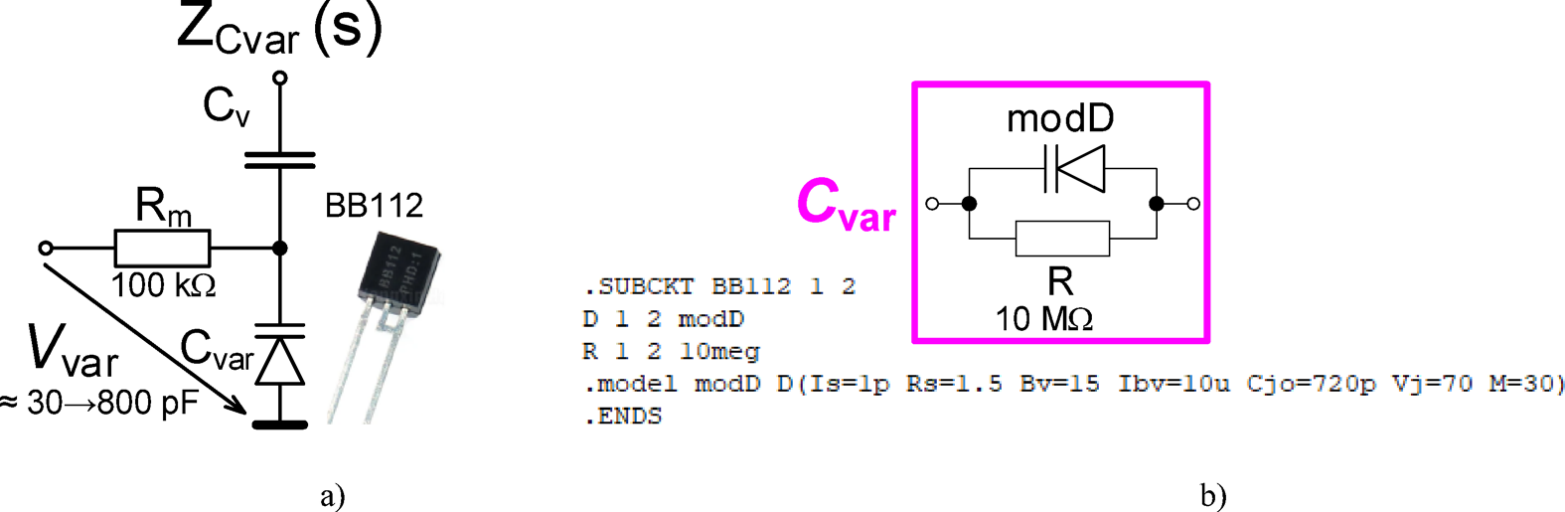
Explanation of varicap: (a) testing circuit of varicap connected as DUT in Fig. 1, (b) model of the varicap.

**Fig. 13:**
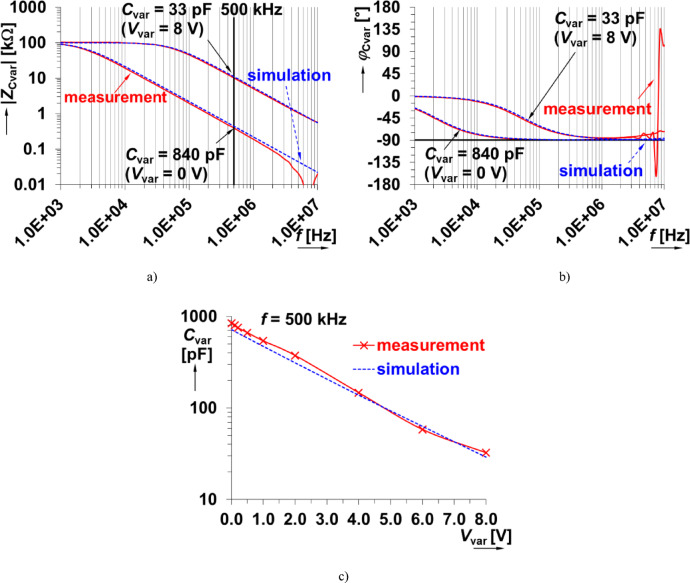
Experimental characteristics of varicap BB112 impedance: (a) impedance magnitude plots, (b) impedance phase plots, (c) dependence of capacitance on reverse bias voltage.

The biasing of this varicap requires additional circuitry, specifically a resistor (*R*_m_) and coupling capacitor (*C*_v_) shown in Fig. 12(a). These additional elements impact the impedance of the overall structure (*Z*_Cvar_(s)). Observing this design, a pole is contributed by *R*_m_ and *C*_var_ at frequency *f*_p_Cvar_ ≅ 1/(2π⋅*R*_m_⋅*C*_var_). This frequency will be within the range from 2 kHz to 50 kHz for *C*_var_ (ranging from 800 pF to 30 pF) when *R*_m_ = 100 kΩ. The impact of *C*_V_ can be significantly below the operational range when an appropriately large value is selected. A value of 10 nF was selected to meet this requirement for the design. To validate the variable capacitance of the varicap with additional circuitry, shown in Fig. 12(a), the impedance magnitude and phase was measured and are shown in Figs. 13(a) and (b), respectively. Measurements were collected using driving voltages (*V*_var_) of 0 V, and 8 V. To evaluate the agreement between experimental data and theoretical expectations, simulations of the design impedance were generated. However, as the BB112 model is not a standard library element or available from the device manufacturer a custom model was required. This model was created using the fundamental definition equation of the model^46–48^: *C*_var_ = *C*_jo_/(1 + *V*_var_/*V*_j_)^M^, where *C*_jo_ is initial zero-bias pn junction capacity, *V*_var_ is reverse bias/driving voltage, *V*_j_ is pn junction potential and *M* is pn junction grading coefficient. The specific model parameters for the PSpice implementation are given in Fig. 12(b). It is important to note that this only describes the varicap operation under reverse biasing (operating as a diode model). The parameters were selected to model the experimental data (not the datasheet information). The simulation values of the impedance magnitude and phase using this model and model parameters are given in Fig. 13 (for boundaries of allowed range of *V*_var_). The difference of expected results and experimental measurement is significantly smaller than in previous MOS-based approach. The agreement supports that the measured varicap does have variable capacitance that aligns with the theoretical definition of this class of device. Overall, the capacitance as a function of the bias voltage using both experimental and simulated data are given in Fig. 13(c), validating the approximate tuning range from 30 pF to 800 pF.

**Fig. 14:**
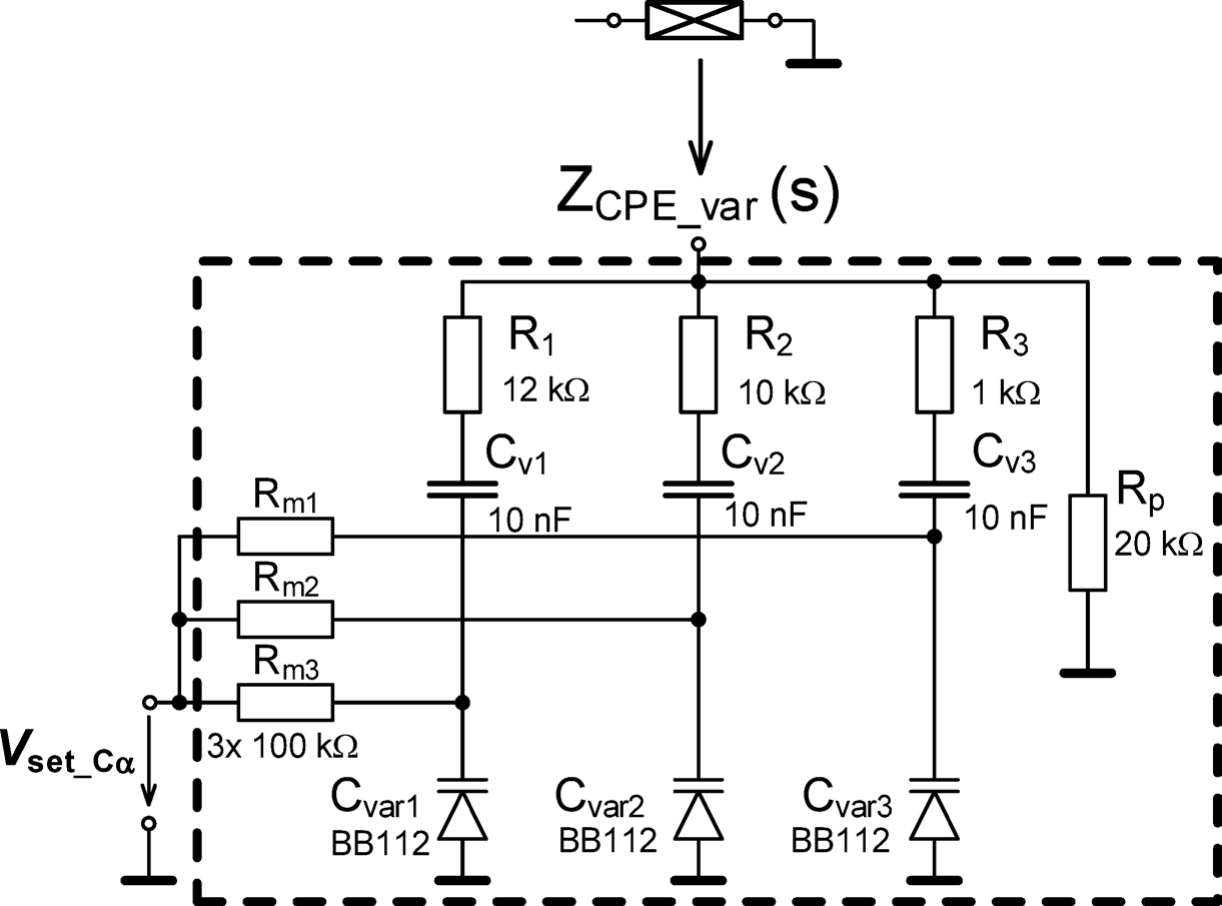
The varicap-based adjustable CPE to realize a half-capacitor with *C*_α_ = 330 nF/sec^0.5^ tunable using a single voltage.

**Fig. 15:**
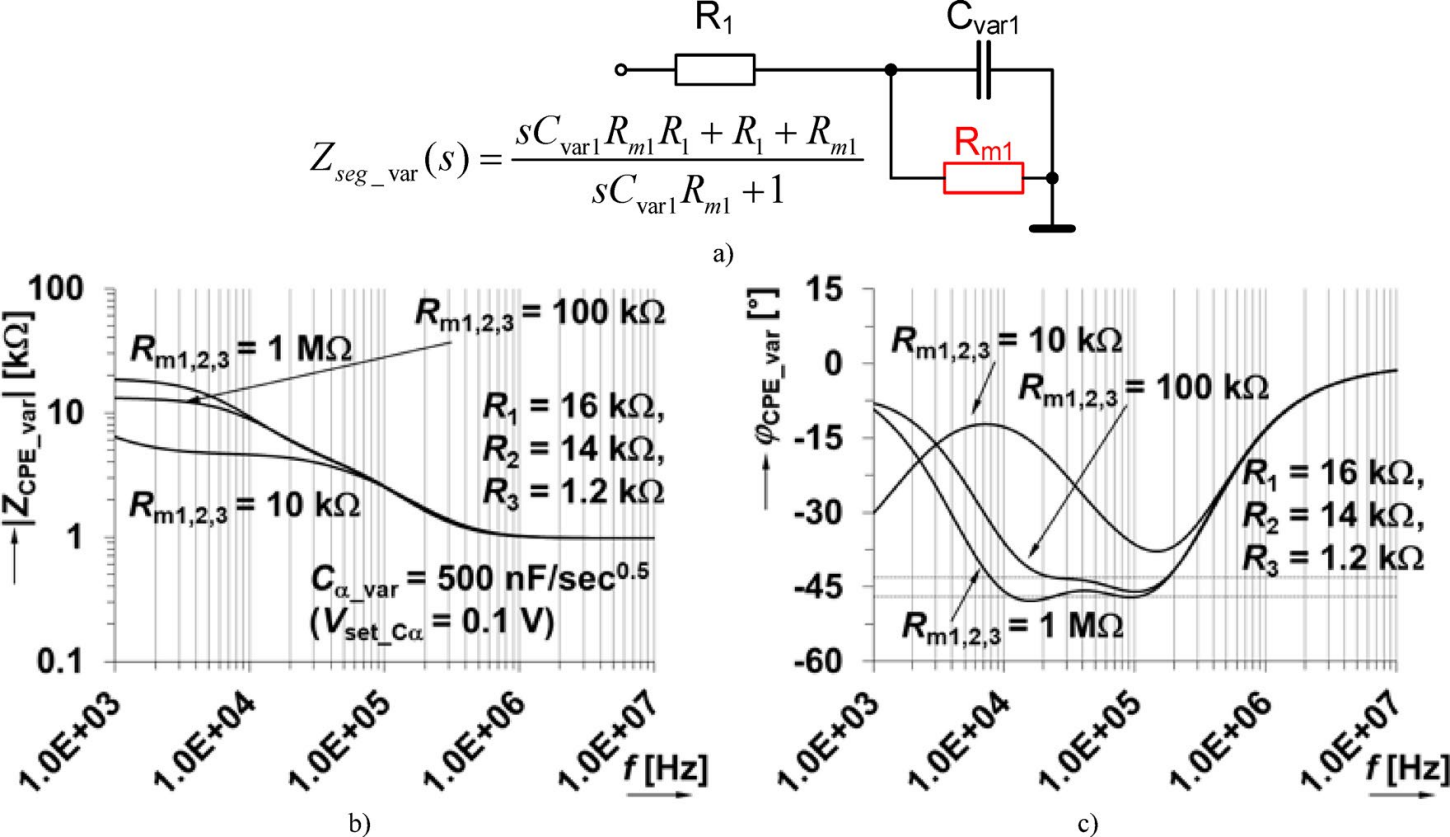
Impact of *R*_m_ value on varicap-based CPE performance: (a) the simplified model of simple CPE segment, (b) impedance magnitude responses of CPE, (c) impedance phase responses of CPE.

The circuit topology of the varicap-based tunable CPE is given in Fig. 14 with the varicaps used as substitutes for the traditional capacitors in each segment. The coupling capacitors and auxiliary resistors are all identical *C*_V1,2,3_ = 10 nF (sufficient for operational bands of tunable CPE above 1 kHz) and *R*_m1,2,3_ = 100 kΩ (feasible in various design technologies). In this case, fixed values of capacitors are set (again in the middle of capacity range performed by varicap) and the optimization tool is used to identify values of resistors *R*_1_ to *R*_p_. The same design criteria are used here for this process (focused on phase ripple at same frequencies as in the previous case: 10 kHz, 30 kHz, 60 kHz, 100 kHz, tolerance defined by Δφ_CPE_ = ± 2°). The value of *C*_α_ is different than the initial theoretical design in Fig. 2 resulting from the (fixed) values of all varicaps *C*_var1,2,3_ being 470 pF, that is available for *V*_var_ = 1 V. The optimization process yielded *R*_1_ = 16.6 kΩ, *R*_2_ = 14.4 kΩ, *R*_3_ = 1.4 kΩ, and *R*_p_ = 20 kΩ. To compensate for the effects of the *R*_m_, modification of *R*_1_-*R*_3_ were implemented to better fit the intended operation band and *C*_α_var_ value. The AC model of a single segment is shown in Fig. 15(a) with impedance given by:

1 $$\:{Z}_{\text{s}\text{e}\text{g}\_\text{v}\text{a}\text{r}}\left(s\right)=\frac{s\:{C}_{\text{v}\text{a}\text{r}1}{R}_{\text{m}1}{R}_{1}+{R}_{1}+{R}_{\text{m}1}}{s{C}_{\text{v}\text{a}\text{r}1}{R}_{\text{m}1}+1}$$

To highlight the impact of *R*_m_ on the zero/pole locations, the impedance magnitude and phase were simulated (for values of 10 kΩ, 100 kΩ, 1 MΩ). These simulations are shown in Fig. 15(b) and (c). From these simulations, it is clear that larger values of *R*_m_ reduce the variations of the segment from its ideal behavior. While using 1 MΩ values would be the most appropriate based on these simulations, this value is challenging to realize for a practical microelectronic implementation. As a compromise, values of 100 kΩ are selected to balance the trade-offs of implementation and performance.

Using these values, the remaining resistors were modified such that: *R*_1_ = 12 kΩ, *R*_2_ = 10 kΩ, *R*_3_ = 1 kΩ, *R*_p_ = 20 kΩ). Also, in this specific design the correction capacitor *C*_p_ has no significant effect (increase of phase outside of operational band). Therefore, *C*_p_ was omitted. The physical realization (photo) of this final design is shown in Fig. 8(b).

**Fig. 16:**
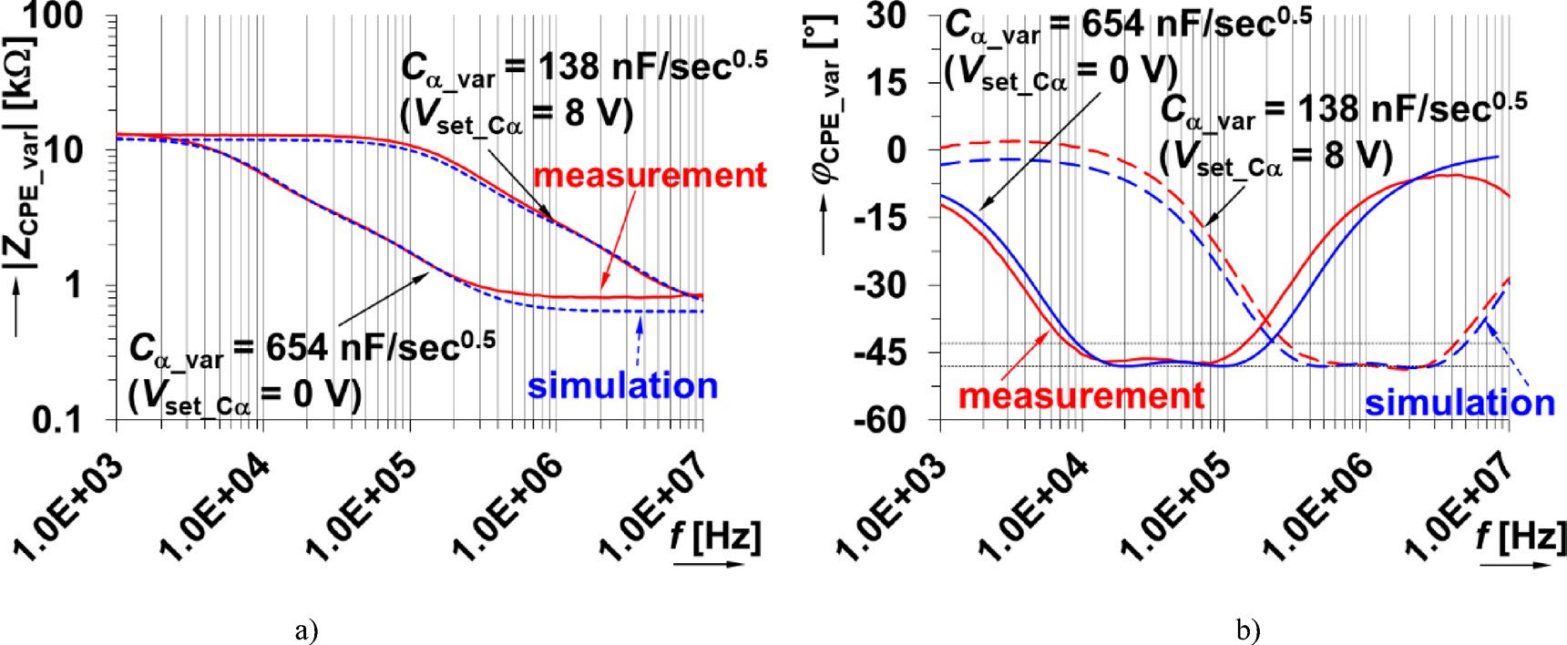
Measured and simulated impedance (a) magnitude and (b) phase of proposed varicap-based adjustable CPE.

**Fig. 17:**
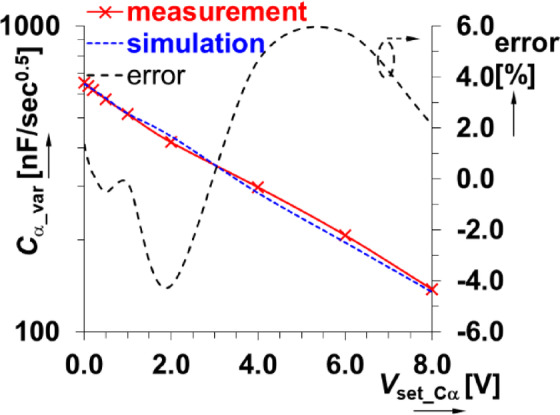
Dependence of the varicap-based CPE pseudocapacitance on the driving voltage.

The impedance magnitude and phase of the physical prototype were measured using the readout setup (shown in Fig. 1 and described in sec3). The cases when *V*_set_Cα_ = 0 V and 8 V are shown in Fig. 16(a) and (b). For comparison, the circuit simulations of the impedance magnitude and phase are also provided. Overall, the experimental results indicate that *C*_α_var_ ranges from 654 nF/sec^0.5^ to 138 nF/sec^0.5^ for *V*_set_Cα_ from 0 V to 8 V. This shows very good agreement with the simulation range of 645 nF/sec^0.5^ to 135 nF/sec^0.5^. The dependence of the pseudocapacitance on the driving voltage is shown in Fig. 17. The center value is approximately *C*_α_var_ = 297 nF/sec^0.5^ (for *V*_set_Cα_ = 4 V) and has an operational bandwidth between 54 kHz and 680 kHz (again, fulfilling the 1 decade goal of this work). This result (*V*_set_Cα_ = 4 V) has been omitted in Fig. 16 for clarity. The examples for the lowest and the highest value of *V*_set_Cα_ are shown.

**Fig. 18:**
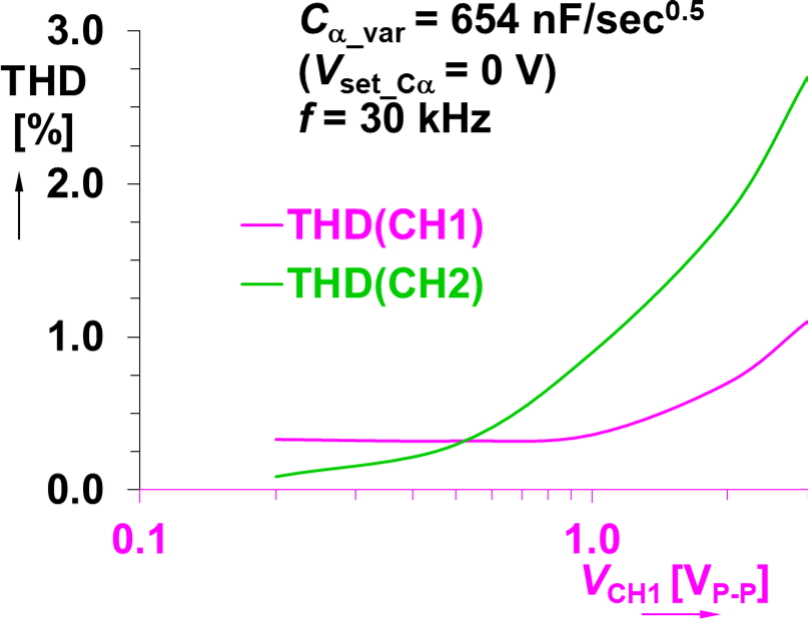
Dependence of THD on voltage levels measured in the experimental setup (Fig. 1) for varicap-based CPE.

The varicaps are typically used in high-frequency (VHF) designs. Therefore, there are no significant deviations from the expected performance at high frequencies (unlike the MOS-based CPE). Similar to the previous design, an analysis of signal distortion (using the same approach previously described) indicates very good performance. The varicap based CPE was tuned to *C*_α_var_ = 654 nF/sec^0.5^ (*V*_set_Cα_ = 0 V) at frequency 30 kHz (the center of operational bandwidth). The corresponding THD results of both channel 1 (voltage across the sensing resistor) and channel 2 (voltage across the CPE) are shown in Fig. 18. The values of THD do not exceed 0.1% for signal levels reaching hundreds of mV (with THD < 1% for signals of approximately 1 V). Therefore, this solution has significantly better linearity when compared with the previous solution.

## Comparison and discussion of both approaches

Overall, this work has presented the design and validation of two approaches to realize an approximated fractional-order capacitor with variable pseudo-capacitance using a single tuning voltage. This design uses the substitution of elements in a traditional RC-ladder approximation with voltage-dependent alternatives; specifically, MOSFET devices and varicaps for variable resistance and capacitance, respectively. Designs were realized using available commercial components to demonstrate feasibility and component values are suitable for further microelectronic implementations. These designs have the potential to support rapid prototyping and design optimization for fractional-order circuits that require fractional-order capacitors.

A comparison of the significant features of this solution compared to existing solutions is provided in Table 2. To summarize, the MOS-based CPE has advantages that include: (a) high sensitivity to driving voltage (very small range of *V*_set_Cα_ is used) and very large adjustability ratio, (b) high adjustability range of *C*_α_ (almost one decade – *C*_α_ can be increased 7×), (c) simple implementation (only capacitors and MOS elements as direct replacements of resistors are required in the topology), (d) design of pseudocapacitances having very high values of *C*_α_ (because of very low *R*_DSon_ – tens, hundreds of Ω). It is important to note the significant disadvantages of the MOSFET-based CPE as well. These include that it is: (a) highly nonlinear with inaccurate dependence of *C*_*α*_ on *V*_set_C*α*_ (differences of almost 20% from expectation, i.e. simulation), (b) no possibility to reach low *C*_*α*_ values (due to *R*_DSon_ range), (c) parasitic device capacitances (*C*_GS_, *C*_G__D_, *C*_DS_) degrade high-frequency operational band (defined operational band of CPE is not fulfilled above 100 kHz for used type of MOS BS170), (d) low THD for only very small signal levels.

The varicap based CPE solves some of the issues of the previous MOS-based solution and has these advantages: (a) better accuracy (error up to 6%) and agreement with theoretical expectations/simulations (attributed to better component tolerances between devices), (b) suitability for high-frequency design (frequency responses are still valid in range above 100 kHz), (c) minimal impact of *C*_α_ tunability on defined operational band (1 decade maintained), (d) the pseudocapacitance C_α_ following initial ideal expectation (Figs. 2, 3 and 4) is achieved, (e) the signal levels up to several hundred of mV can be applied with acceptable or very low distortion. The disadvantages of this design are that it has: (a) slightly reduced range of *C*_α_ adjustability (*C*_α_ can be increased 5x) in comparison to MOS-based CPE, (b) quite large range of required driving voltage *V*_set_Cα_ (0→8 V), (c) increased complexity of circuit topology (auxiliary elements as *R*_m_ and coupling capacitors *C*_V_).

**Table 2:** Comparison of proposed designs of single voltage adjustable cpes.

Solution	MOS-based	Varicap-based
Approximate operational frequency range of CPE (*C*_α_varied and valid in 1 frequency decade)	500 Hz→100 kHz	8 kHz→5 MHz
Driving voltage *V*_set_Cα_	2.03→2.2 V	0→8 V
Simulated *C*_α_ range	17→107 µF/sec^0.5^	645→135 nF/sec^0.5^
Measured *C*_α_ range	15→109 µF/sec^0.5^	654→138 nF/sec^0.5^
Ratio of max and min *C*_α_(*C*_α_max_ / *C*_α_min_)	7	5
Maximal *C*_α_ error in adjustable range	−19%	−6%
Accuracy	Low	High
Measured adjustability ratio [(*C*_α_max_ / *C*_α_min_)/(*V*_set_Cα_max_ / *V*_set_Cα_min_)]	6.7	< 1 when *V*_set_Cα_ → 0
Approximate maximally processed voltage without significant signal shape distortion	100 mV_P−P_	units of V_P−P_
Maximal THD in measuring setup (*V*_CH1_ = 100 mV_P−P_)	5.5%	0.1%
The lowest achieved THD in the measuring setup	0.6% (*V*_CH1_ = 20 mV_P−P_)	0.1% (*V*_CH2_ = 80 mV_P−P_)

Each of the presented solutions has the potential to support different applications or implementations. The MOS-based solution has the potential to be integrated into microelectronic scale designs. The integrated implementation supposes operation with very low signal levels (so the THD of this design may not significantly effect its performance for these applications). Similarly, *R*_DSon_ can be influenced by specific design of MOS elements (aspect ratio width/length) or the resistor of CPE can be created by different way (several MOS elements^49^, special active element as linear transconductance amplifier^50^, etc.), providing greater design flexibility with an integrated design than was possible using a single commercial element. On the other hand, the design of adjustable capacitances (of large values especially) is challenging and limited for integrated designs (chip area requirements are high). Also, discrete design of large *C*_α_ values (tens of µF) is typical for discrete MOS-based CPE. The varicap-based CPE is more suitable for discrete circuit design expecting linear processing of higher signal levels and high accuracy.

While this work has demonstrated the feasibility of this design approach, there are a few limitations which require future investigation. This work was limited to a device with half-order (*α* = 0.5) and further investigation to evaluate this approach for the range of fractional orders (0 < *α <* 1*)* is needed. Similarly, further work is needed to evaluate the feasibility of using this approach to extend the operating frequency range beyond one decade which would increase the usability of devices designed with this method. A limitation of the current design approach is the requirement to adjust component values based on the characteristics of the substitution element. Future algorithms could be improved by including these tuning steps into the design procedure conducted with the simulation tools and their available models. Finally, these designs only investigated one type of MOSFET (BS170) and vari-cap (BB112) device, which may limit the performance range due to the specific characteristics of each. Future work should investigate the voltage-dependent resistance and capacitance of other commercially available components to determine if there are available devices that can improve the accuracy of these designs (or extend their operating ranges).

## Conclusion

Our work has demonstrated two methods to design a constant phase element with variable pseudocapacitance controlled by a single driving DC voltage. The design targets for this work were to achieve a single frequency decade operability with phase ripple of ± 2°. We obtained two ranges of pseudocapacitance (15µF/sec^0.5^ to 109 µF/sec^0.5^ and 138 nF/sec^0.5^ to 654 nF/sec^0.5^) adjusted by DC driving voltage in two solutions of CPE using standard discrete elements (resistors or capacitors) and electronically adjustable equivalents of these elements (MOSFET transistors and varicaps, respectively). The obtained ranges of pseudocapacitance are very close to the design intentions. The MOS-based approach has a wider adjustability range but is limited to low amplitude signals but has greater potential for integration as a simple and compact integrated circuit. The operational frequency range for the MOS-based solution spans from 4.6 kHz to 49 kHz, while for the varicap-based solution it ranges from 54 kHz to 680 kHz (both ranges valid for center value of pseudocapacity, i.e. the valid operational frequency decade/bandwidth is located in the middle of possible frequency positions). Both solutions were designed to cover one decade of frequency. The full frequency range where the operational bands can be shifted by pseudocapacitance variation (still valid in 1 decade) falls between 500 Hz and 100 kHz and between 8 kHz and 5 MHz. Overall, the experimental measurements confirmed an acceptable match with the designed parameters. While this work demonstrates the feasibility of this approach, the driving goal of pseudocapacitance adjustment requires further research focused on design approaches, algorithms and methodologies (fixation of values of specific elements for their simultaneous adjustment), i.e. modification of algorithms serving for the design of fractional-order RC approximants to address the current limitations. The electronically adjustable CPE, controlled by DC voltage, offers a significant advantage in the design of electronically adjustable fractional-order circuits and systems. This adjustability is particularly important for modifying the time constant, which directly impacts key parameters of various circuit applications, such as passive and active filters, phase shifters, and oscillators. Modern analog electronics increasingly demand designs that incorporate electronically controlled (by DC voltage) parameters of devices, enabling simple and seamless adjustment via computer or digital systems. This allows important analog systems parameters, such as cut-off frequencies, gains, and others, to be easily modified or automatically adapted to chaining conditions. There are various unsolved issues in the field of passive and active adjustable CPEs (implementation of active devices bringing the feature of adjustability, increasing the ranges of adjustability, improvements of linearity, changing the trends and dependences of pseudocapacitance on control parameters, solving the aspects of the signal distortion (passive vs. active), designing the circuits with simple single-parameter adjustability of the order/phase, etc.). These issues deserve further research efforts and our research opens some of them.

## Data Availability

Key data of all results is provided within the manuscript. Supplementary data (datasets of computer-aided analysis of circuits and detailed measurement results) can be shared with readers based on their reasonable request (addressed to corresponding author).

## Abbreviations

a: Order of the CPE
C_DS_: Drain source capacitance of the MOS transistor
C_jo_: Initial zero-bias pn junction capacity
C_p_: Parasitic (stray) capacity
CPE: Constant phase element
C_v_: Decoupling capacity
C_a_: Pseudocapacitance (initial/nominal general RC prototype)
C_a_MOS_: Pseudocapacitance of CPE using MOS transistors
C_a_var(s)_: Pseudocapacitance of CPE using varicaps
DC: Direct current
g_fs_: Transconductance of MOS transistor
M: pn junction grading coefficient
MOS, MOSFET: Metal oxide silicium transistor
PSpice: Personal Simulation Program with Integrated Circuit Emphasis
RC, R or C: Resistor, capacitor
R_DSon_: Small signal on resistance of the MOS transistor
R_m_: Auxiliary resistor for varicap adjustment
THD: Total harmonic distortion
varicap: Variable capacitance diode
V_CH1,2_: Voltage of channel 1,2 of the oscilloscope inputs
V_GS_: Gate source voltage of MOS transistor
VHF: Very high frequency
V_j_: pn junction potential
V_set_Ca_: Driving DC voltage for adjustability of CPE
V_th_: Threshold voltage of MOS transistor
V_var_: Reverse bias voltage for varicap capacity adjustment
Z_CPE(s)_: Impedance of the general CPE device (RC prototype)
Z_CPE_MOS(s)_: Impedance of the CPE using MOS transistors
Z_CPE_var(s)_: Impedance of the CPE using varicaps
Z_Cvar(s)_: Impedance of the varicap
Z_DSon(s)_: Impedance of the MOS transistor

## References

[1] Elwakil, A. S. Fractional-order circuits and systems: an emerging interdisciplinary research area. *IEEE Circuits Syst. Mag.* **10** (4), 40–50. 10.1109/MCAS.2010.938637 (2010).

[2] Freeborn, T. J. A survey of fractional-order circuit models for biology and biomedicine. *IEEE J. Emerg. Selected Top. Circuits Syst.* **3** (3), 416–424. 10.1109/JETCAS.2013.2265797 (2013).

[3] Podlubny, I. *Fractional Differential Equations, First Edition Ed* (Elsevier, 1999).

[4] Vastarouchas, C., Tsirimokou, G. & Psychalinos, C. Extraction of Cole-Cole model parameters through low-frequency measurements. *AEU-Int J. Electron. Commun.* **84**, 355–358. 10.1016/j.aeue.2017.11.020 (2018).

[5] Vastarouchas, C., Psychalinos, C., Elwakil, A. S. & Al-Ali, A. A. Novel two-measurements-only Cole-Cole bio-impedance parameters extraction technique. *Measurement* **131** (1), 394–399. 10.1016/j.measurement.2018.09.008 (2019).

[6] Ibba, P. et al. Bio-impedance and circuit parameters: an analysis for tracking fruit ripening. *Postharvest Biol. Technol.* **159**, 1–8. 10.1016/j.postharvbio.2019.110978 (2020).

[7] Lopes, A. M., Machado, J. A. T. & Ramalho, E. On the fractional-order modeling of wine. *Eur. Food Res. Technol.* **243**, 921–929. 10.1007/s00217-016-2806-x (2017).

[8] Pelc, D., Marion, S. & Basletić, M. Four-contact impedance spectroscopy of conductive liquid samples. *Rev. Sci. Instrum.* **82**, 1–5. 10.1063/1.3609867 (2011). 10.1063/1.3609867 21806199

[9] Slay, J. et al. Distinguishing liquid solutions with alcohol using electrical impedance measurements: preliminary study for food safety applications. *IEEE Sens. J.* **23** (22), 26997–27007. 10.1109/JSEN.2023.3315798 (2023).

[10] Islam, M., Wahid, K. A., Dinh, A. V. & Bhowmik, P. ‘Model of dehydration and assessment of moisture content on onion using EIS’. *J. Food Sci. Technol.* **56** (6), 2814–2824. 10.1007/s13197-019-03590-3 (2019). 31205337 10.1007/s13197-019-03590-3 PMC6542975

[11] Patil, A. C., Fernandez la Villa, A., Mugilvannan, A. K. & Elejalde, U. Electrochemical investigation of edible oils: experimentation, electrical signatures, and a supervised learning–case study of adulterated peanut oils. *Food Chem.* **402** (15), 1–13. 10.1016/j.foodchem.2022.134143 (2023). 10.1016/j.foodchem.2022.134143 36148762

[12] Tapadar, A. & Adhikary, A. High Sensitivity Milk Adulteration Detector Using Fractional Order Colpitts Oscillator, 2023 IEEE Conference on AgriFood Electronics (CAFE), Torino, Italy, pp. 85–88. 10.1109/CAFE58535.2023.10292088 (2023).

[13] Sanchez, B., Li, J., Geisbush, T., Bardia, R. B. & Rutkove, S. B. ‘Impedance alterations in healthy and diseased mice during electrically induced muscle contraction’. *IEEE Trans. Biomed. Eng.* **63** (8), 1602–1612. 10.1109/TBME.2014.2320132 (2016). 24800834 10.1109/TBME.2014.2320132

[14] Prasad, A. & Roy, M. ‘Bioimpedance analysis of vascular tissue and fluid flow in human and plant body: A review. *’ Biosyst Eng.* **197**, 170–187. 10.1016/j.biosystemseng.2020.06.006 (2020).

[15] Fu, B. & Freeborn, T. J. ‘Cole-impedance parameters representing biceps tissue bioimpedance in healthy adults and their alterations following eccentric exercise,’’ J. *Adv. Res.* **25**, 285–293. 10.1016/j.jare.2020.05.016 (2020). 10.1016/j.jare.2020.05.016 PMC7474209 32922994

[16] Wohlgemuth, K. J., Freeborn, T. J., Southall, K. E., Hare, M. M. & Mota, J. A. Can segmental bioelectrical impedance be used as a measure of muscle quality? *Med. Eng. Phys.* **124**, 1–10. 10.1016/j.medengphy.2024.104103 (2024). 10.1016/j.medengphy.2024.104103 38418031

[17] AboBakr, A., Said, L. A., Madian, A. H. & Elwakil, A. S. Radwan,‘‘Experimental comparison of integer/fractional-order electrical models of plant’’. *AEU-Int J. Electron. Commun.* **80**, 1–9. 10.1016/j.aeue.2017.06.010 (2017).

[18] Kapoulea, S., Psychalinos, C. & Elwakil, A. S. Realization of Cole-Davidson function-based impedance models: application on plant tissues. *Fractal Fract. J.* **4** (4), 1–15. 10.3390/fractalfract4040054 (2020).

[19] Panagiotis Bertsias, S., Kapoulea, C., Psychalinos & Elwakil, A. S. A collection of interdisciplinary applications of fractional-order circuits. *Elsevier eBooks*. 35–69. 10.1016/b978-0-12-824293-3.00007-7 (2022).

[20] Stavroula Kapoulea, C., Psychalinos & Elwakil, A. S. Power law filters: A new class of fractional-order filters without a fractional-order laplacian operator. *AEU - Int. J. Electron. Commun.* **129**, 153537–153537. 10.1016/j.aeue.2020.153537 (2021).

[21] Nako, J., Psychalinos, C., Elwakil, A. S. & Jurisic, D. Design of Higher-Order fractional filters with fully controllable frequency characteristics. *IEEE Access* **11**, 43205–43215. 10.1109/ACCESS.2023.3271863 (2023).

[22] Nako, J., Psychalinos, C., Elwakil, A. S. & Minaei, S. Non-integer order generalized filters designs. *IEEE Access* **11**, 116846–116859. 10.1109/ACCESS.2023.3325911 (2023).

[23] Nako, J., Psychalinos, C. & Elwakil, A. S. One active element implementation of fractional-order Butterworth and Chebyshev filters. *AEU - Int. J. Electron. Commun.* **168**, 154724–154724. 10.1016/j.aeue.2023.154724 (2023).

[24] Nako, J., Psychalinos, C., Khateb, F. & Elwakil, A. S. Bilinear double-order filter designs and application examples. *IEEE Access* **12**, 14040–14049. 10.1109/ACCESS.2024.3357092 (2024).

[25] Sotner, R. et al. On the performance of electronically tunable fractional-order oscillator using grounded resonator concept. *AEU - Int. J. Electron. Commun.* **129**, 153540–153540. 10.1016/j.aeue.2020.153540 (2021).

[26] Varshney, G., Pandey, N. & Pandey, R. Design and implementation of OTA based fractional-order oscillator. *Analog Integr. Circuits Signal Process.* **113** (1), 93–103. 10.1007/s10470-022-02069-0 (2022).

[27] Tapadar, A., Sachan, S. & Adhikary, A. Complete design guidelines for Fractional-Order colpitts oscillator with Non-ideal Op-Amp. *Circuits Syst. Signal. Process.* **41**, 5340–5365. 10.1007/s00034-022-02045-z (2022).

[28] Tavazoei, M. S. Frequency content preservation in fractional Multi-Frequency oscillators despite reducing the number of energy storage elements. *Circuits Syst. Signal. Process.* **41**, 3066–3080. 10.1007/s00034-021-01944-x (2022).

[29] Tavazoei, M. S. Closed-form oscillatory condition in electrical circuits containing two fractional order elements. *IEEE Trans. Circuits Syst. II: Express Briefs* **69** (6), 2687–2691. 10.1109/TCSII.2022.3149857 (2022).

[30] Mohapatra, A. S., Zimmermann, S. & Biswas, K. Designing fractional oscillator for sensing different types of lossy capacitors. *IEEE Trans. Instrum. Meas.* **72**, 1–9. 10.1109/TIM.2023.3264030 (2023).37323850

[31] Sivarama Krishna, M., Das, S., Biswas, K. & Goswami, B. Fabrication of a fractional order capacitor with desired specifications: A study on process identification and characterization. *IEEE Trans. Electron. Devices*. **58** (11), 4067–4073. 10.1109/TED.2011.2166763 (2011).

[32] Mondal, D. & Biswas, K. Performance study of fractional order integrator using single-component fractional order element. *IET Circuits Devices Syst.* **5** (4), 334–342. 10.1049/iet-cds.2010.0366 (2011).

[33] Adhikary, A., Khanra, M., Sen, S. & Biswas, K. Realization of a carbon nanotube based electrochemical fractor, 2015 IEEE International Symposium on Circuits and Systems (ISCAS), Lisbon, Portugal, 2329–2332. 10.1109/ISCAS.2015.7169150 (2015).

[34] Ushakov, P., Shadrin, A., Kubanek, D. & Koton, J. Passive fractional-order components based on resistive-capacitive circuits with distributed parameters, 39th International Conference on Telecommunications and Signal Processing (TSP), Vienna, Austria, 2016, 638–642. 10.1109/TSP.2016.7760960 (2016).

[35] Petrzela, J. Accurate constant phase elements dedicated for audio signal processing. *Appl. Sci.* **9** (22), 1–38. 10.3390/app9224888 (2019).

[36] Kapoulea, S., Psychalinos, C. & Elwakil, A. S. Single active element implementation of fractional-order differentiators and integrators. *AEU Int. J. Electron. Communication*. **97** (12), 6–15. 10.1016/j.aeue.2018.09.046 (2018).

[37] Valsa, J., Dvořák, P. & Friedl, M. Network model of the CPE. *Radioengineering* **20** (3), 619–626 (2011).

[38] Valsa, J. & Vlach, J. RC models of a constant phase element. *Int. J. Circuit Theory Appl.* **41** (1), 59–67. 10.1002/cta.785 (2013).

[39] Sotner, R. et al. Synthesis and design of constant phase elements based on the multiplication of electronically controllable bilinear immittances in practice. *AEU - Int. J. Electron. Commun.* **78**, 98–113. 10.1016/j.aeue.2017.05.013 (2017).

[40] Sotner, R., Polak, L., Jerabek, J. & Petrzela, J. Simple two operational transconductance amplifiers-based electronically controllable bilinear two Port for fractional-order synthesis. *Electron. Lett.* **54**, 1164–1166. 10.1049/el.2018.5575 (2018).

[41] Prommee, P. & Pienpichayapong, P. Reconfigurable Fractional-Order operator and bandwidth expansion suitable for PIα controller. *IEEE Trans. Industr. Electron.* **71** (5), 5126–5136. 10.1109/TIE.2023.3288170 (2024).

[42] Mijat, N., Jurisic, D. & Moschytz, G. S. Analog modeling of Fractional-Order elements: A classical circuit theory approach. *IEEE Access.* **9**, 110309–110331. 10.1109/ACCESS.2021.3101160 (2021).

[43] Sotner, R., Domansky, O., Langhammer, L. & Petrzela, J. Comparison of Simple Design Methods for Voltage Controllable Resistance, 2020 30th International Conference Radioelektronika (RADIOELEKTRONIKA), Bratislava, Slovakia, 1–6. 10.1109/RADIOELEKTRONIKA49387.2020.9092366 (2020).

[44] BS170 / MMBF170 -N-Channel Enhancement Mode Field Effect Transistor BS170 / MMBF170 N-Channel Enhancement Mode Field Effect Transistor General Description. Accessed: Dec. 07, 2024. [Online]. Available: https://img.gme.cz/files/eshop_data/eshop_data/2/213-008/dsh.213-008.2.pdf (2010).

[45] Siemens Silicon Variable Capacitance Diode BB112. [online] Available: https://datasheetspdf.com/pdf/493437/SiemensGroup/BB112/1. Accessed December 07 2024 (2024).

[46] Standard.dio. - LTwiki-Wiki for LTspice, ltwiki.org. https://ltwiki.org/index.php?title=Standard.dio

[47] Models, S. P. I. C. E. & Rectifiers Diodes and Electronics Textbook, www.allaboutcircuits.com. https://www.allaboutcircuits.com/textbook/semiconductors/chpt-3/spice-models/

[48] Spice Modell für BB112, Mikrocontroller.net, Aug. 07. (2013). https://www.mikrocontroller.net/topic/304813. Accessed December 07 2024 (2013).

[49] Yucel, F. & Yuce, E. A new electronically fine tunable grounded voltage controlled positive resistor. *IEEE Trans. Circuits Syst. II: Express Briefs* **65** (4), 451–455. 10.1109/TCSII.2017.2702740 (2018).

[50] Arbel, A. F. & Goldminz, L. Output stage for current-mode feedback amplifiers, theory and applications. *Analog Integr. Circ. Sig Process.* **2**, 243–255. 10.1007/BF00276637 (1992).

